# The Regulatory Capacity of Bivalent Genes—A Theoretical Approach

**DOI:** 10.3390/ijms18051069

**Published:** 2017-05-17

**Authors:** Torsten Thalheim, Maria Herberg, Markus Loeffler, Joerg Galle

**Affiliations:** 1 Interdisciplinary Centre for Bioinformatics, University Leipzig, Haertelstr. 16-18, 04107 Leipzig, Germany; thalheim@izbi.uni-leipzig.de (T.T.); herberg@izbi.uni-leipzig.de (M.H.); 2 Institute for Medical Informatics, Statistics and Epidemiology, University Leipzig, Haertelstr. 16-18, 04107 Leipzig, Germany; markus.loeffler@imise.uni-leipzig.de

**Keywords:** bivalent gene, histone modification, gene expression heterogeneity, lineage specification, aberrant DNA methylation, blast formation

## Abstract

Bivalent genes are frequently associated with developmental and lineage specification processes. Resolving their bivalency enables fast changes in their expression, which potentially can trigger cell fate decisions. Here, we provide a theoretical model of bivalency that allows for predictions on the occurrence, stability and regulatory capacity of this prominent modification state. We suggest that bivalency enables balanced gene expression heterogeneity that constitutes a prerequisite of robust lineage priming in somatic stem cells. Moreover, we demonstrate that interactions between the histone and DNA methylation machineries together with the proliferation activity control the stability of the bivalent state and can turn it into an unmodified state. We suggest that deregulation of these interactions underlies cell transformation processes as associated with acute myeloid leukemia (AML) and provide a model of AML blast formation following deregulation of the Ten-eleven Translocation (TET) pathway.

## 1. Introduction

In the last decade, different histone modifications have been implicated in transcriptional regulation of genes. In this regard, tri-methylation of lysine 4 and 27 at histone H3 are well studied modifications [1]. Tri-methylation of lysine 4 at histone H3 (H3K4me3), if present at nucleosomes that are associated with CpG-rich gene promoters, has been found to be positively correlated with the transcription of the respective gene. In contrast, tri-methylation of lysine 27 at histone H3 (H3K27me3), if present at these nucleosomes, is often associated with gene repression. Experimentally, a non-linear, approximately sigmoidal relationship between gene transcription and the modification level of the gene promoter has been observed for both the H3K4me3 and the H3K27me3 modification [2]. Thereby, the modification level of H3K4me3 at the promoter of a gene changes from low to high, when the transcription rate of the gene exceeds a particular threshold. The opposite holds true for the modification level of H3K27me3.

It has been shown that both the H3K4me3 and the H3K27me3 modification are set by specific methyltransferase complexes (tri-thorax and polycomb group complexes, respectively) that can not only write but also read the modification [1]. This capability results in a positive feedback on writing the modification. Such feedback enables bi-stability, i.e., the coexistence of two stable modification states. Accordingly, cells can either carry a modified or an unmodified gene under the same, defined conditions. We have predicted that the occurrence of bi-stable modification states depends, e.g., on the histone (de)-modification rates, the methylation of the associated DNA or the number of nucleosomes that are cooperatively modified [3].

Genes that carry both the H3K4me3 and the H3K27me3 modification at nucleosomes associated with their promoter region are called bivalent and typically show an intermediate gene expression. Initially, bivalency has been described as a “zonal” phenomenon only, in which a broad domain of H3K27me3 surrounds a narrower one of H3K4me3. However, it has been demonstrated recently that both modifications indeed can reside at the same nucleosome [4]. We here focus on such truly bivalent nucleosomes being often enriched in the vicinity of the transcription start site of a gene [5]. Bivalent genes have come into research focus because they often change their modification state from bivalent to monovalent during developmental processes [6]. These dynamic state changes are accompanied by alterations in the transcription of the affected gene. Loss of H3K27me3 is accompanied by transcriptional activation of the gene, while a loss of H3K4me3 is related to its transcriptional repression. Well known examples of bivalent genes are genes encoding transcription factors (TFs) involved in developmental and stem cell differentiation processes, such as *GATA3* [7] and Achaete-scute complex homolog 2 (*ASCL2*) [8] being involved in hematopoietic and intestinal lineage specification, respectively, or *ATOH1* controlling sensory hair cell differentiation [9]. Findings on such TFs suggest an instructive role of bivalent histone modifications regarding cell fate decisions by controlling heterogeneous gene expression. Nevertheless, a mechanistic understanding of this control process is still largely missing.

In the following, we introduce a theoretical model of bivalency to gain a mechanistic understanding of the link between histone modification and transcriptional regulation. In particular, we analyze the stability of the bivalent state depending on the properties of the histone modification and transcriptional machinery. By computational simulation, we demonstrate: (i) that the regulatory circuit can induce stable gene expression heterogeneity in proliferating cell populations by (reversible) transitions from the bivalent modification state into the monovalent states; and (ii) that these transitions are a consequence of a particular kind of asymmetric cell division randomly distributing modified histones from the mother onto the daughter cells. Subsequently, we investigate the role of DNA methylation in this process. We show: (i) that following DNA methylation the bivalent state becomes destabilized; and (ii) that under these conditions gene expression heterogeneity can be induced by active DNA demethylation. Our findings have implications for a better understanding the control and deregulation of cell population heterogeneity as observed, e.g., in the hematopoietic stem cell system and during tissue transformation, as e.g., blast formation in acute myeloid leukemia (AML).

## 2. Basic Model Assumptions

### 2.1. Epigenetic Regulation of Transcription: The Basic Regulatory Circuit

In the following, we built on an established theoretical model of histone methylation, which has been introduced by our group previously [3,10]. This model, which applies to histone modifications of CpG-rich gene promoters, considers dynamic histone modification states due to permanent histone de-modification processes. It quantifies modification levels as the fraction of modified nucleosomes associated with the promoter. The modification processes are described in dependence of binding properties of the histone methyltransferase (HMT) complexes to nucleosomes and the associated DNA, which change depending on their respective methylation state. Details on the basic model system can be found in [3].

In an extension of the model [11], we have recently introduced a positive feedback loop between the transcriptional activity of the gene (T) and the H3K4me3 modification level (m_4_). Based on experimental findings, we assumed that transcriptional activity facilitates recruitment of H3K4me3 HMTs [12] and that H3K4me3 contributes in recruiting Pol II [13]. In the following, we assume a negative feedback loop between transcriptional activity (T) and the H3K27me3 level (m_27_) in addition. We assume that transcriptional activity suppresses the recruitment of the H3K27me3 HMTs and that H3K27me3 impedes recruitment of Pol II. In fact, binding of activating TFs in the promoter region has been reported as a major factor suppressing H3K27me3 modification [14]. Moreover, polycomb group complexes, containing the HMTs of H3K27me3, have been suggested to suppress transcription by preventing the binding of acetyl-transferases to target genes [15].

**Figure 1:**
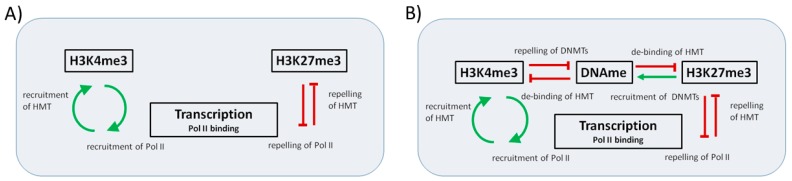
Sketch of the interactions within the regulatory circuit. (**A**) The simplified model version MV1 focuses on the interaction between histone modification and transcription, while effects of DNA methylation are neglected. We consider a positive feedback (green arrows) between H3K4me3 and transcription and a negative one (red T bars) between H3K27me3 and transcription; (**B**) In an extended model version MV2, both histone modifications are considered to be suppressed by DNA methylation that weakens binding of the respective HTMs. H3K4me3 suppresses DNA methylation by suppressing DNA methyltransferase (DNMT) recruitment, while H3K27me3 recruits DNMTs.

In a first version of the model (MV1, Figure 1A), we neglect effects of DNA methylation at the gene promoter. We describe genes that are effectively protected from DNA methylation independent of the histone modification states of the associates nucleosomes; as genes that undergo permanent DNA demethylation, e.g., due to the activity of Ten-eleven Translocation (TET) proteins (reviewed in [16]). We do not consider explicit interdependencies between the two histone modifications.

This model constraint will be released in a second version of the model (MV2, Figure 1B) that includes interactions between histone and DNA methylation at the gene promoter. In order to describe the DNA methylation level of the gene promoter m_DNA_, i.e., the fraction of methylated CpGs at the promoter, we basically use the same mathematical formulation of the DNA methylation machinery as described in [10]. Thus, we consider maintenance and de novo DNA methylation, controlled by the DNA methyltransferases 1 (DNMT1) and 3a,b (DNMT3a,b), respectively. Both processes are assumed to be active during cell division only. In accordance with experimental observations, we assume DNA methylation to be subject to regulatory feedback loops with both H3K4me3 and H3K27me3. We assume that H3K4me3 suppresses binding of de novo DNMTs [17] and that DNA methylation weakens the binding of H3K4me3 HMTs to DNA [18]. The latter is assumed also for H3K27me3 HMTs [14]. In contrast to H3K4me3, H3K27me3 is assumed to contribute in recruiting de novo DNMTs, and thus destabilizes itself. This is in agreement with experimental findings showing that the H3K27me3 HMT Enhancer of zeste homolog 2 (EZH2) recruits DNMTs, but the DNMTs subsequently do not induce de novo DNA methylation before H3K27me3 is removed [19].

DNA methylation is assumed to have no direct impact on transcription. This assumption is in agreement with experimental findings, which show that transcriptional silencing of promoters precedes their DNA methylation [20].

### 2.2. Simulations of Cell Populations

We analyze the dynamics of transcription, histone modifications and DNA methylation in a small cell population of 100 cells by stochastic simulations of the behavior of the bivalent circuit. One simulation consists of 10,000 simulation steps. We record the state of the cells every 100 simulation steps, here referred as one time step. Thereby, changes of the regulatory state of an individual cell originate in fluctuations of the histone methylation states that depend on the methylation state of the associated DNA and on gene transcription. We neglect all other types of permanent fluctuations, e.g., fluctuations in Pol II binding [21]. Fluctuations of histone modifications are simulated considering modification and de-modification of individual histones that are associated with the gene promoter with rate k_mod_ θ^K^ and k_de_, respectively. Here, k_mod_ and k_de_ are constants and θ^K^ (K = 4, 27) is the binding affinity of the respective HMT [3]. Subsequent changes in DNA methylation and transcription are simulated according to differential equations given in the text below. The regulatory states are mapped on discrete cell fates (e.g., the bivalent state) that define subpopulations of cells.

It has been shown experimentally that during cell division the histones of the mother cell are randomly distributed onto the two daughters [22]. Accordingly, cell division results in a strong dilution of the modified histones in both daughter cells. We simulated these effects of cell division by performing a dilution of the modified histones after a (gamma-distributed) waiting time, i.e., after cell cycle time τ [23]. Whenever one daughter cell retains a fraction x of the modified nucleosomes present in the mother, the other daughter retains a fraction (1 − x). We consider that the cells compete for space within their niches. Accordingly, we keep the total number of cells fixed assuming that each increase due to a cell division event is exactly balanced by cell loss from the niche. Thereby, subpopulations might differ in their specific cell loss probability.

## 3. Results

### 3.1. The Occurrence of the Bivalent Modification States

**Figure 2:**
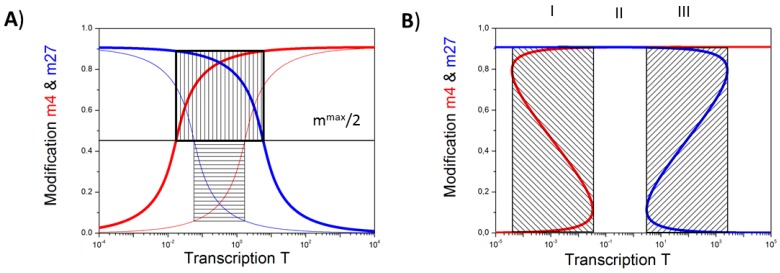
Occurrence of the bivalent state: (**A**) Shown are the H3K4me3 (red) and H3K27me3 (blue) modification levels for different binding efficiencies of the HMTs. Intersections with the black line m^max^/2 define T_K_ (K = K4, K27). For T_K27_ < T_K4_ (System A1, thin lines) an unmodified state is associated with transcription levels T_K27_ < *T* < T_K4_ (box with horizontal lines), while for T_K27_ > T_K4_, (System A2, thick lines) a bivalent state is associated with transcription levels T_K4_ < *T* < T_K27_ (box with vertical lines); (**B**) Increasing the number of cooperative nucleosomes at the promoter, here for system A1, enforces bistable modification states (red: H3K4me3, blue: H3K27me3). They can occur as exclusive solutions (range II) or can co-occur with one of the monovalent states (ranges I: K27 and III: K4). These states are hard to measure experimentally as averages over many cells will pretend intermediate modification levels.

Our model is consistent with the experimental finding of a non-linear, approximately sigmoidal relationship between gene transcription and the modification level of the gene promoter; for both the H3K4me3 and the H3K27me3 modification [2]. The H3K4me3 (H3K27me3) modification level of a gene changes from low to high (high to low), when its transcription rate exceeds a particular threshold. Depending on the transcriptional level at which this change occurs for the individual modification, here referred as T_K_ (K = K4, K27), one can, in general, distinguish two cases (Figure 2A).

In the case T_K27_ is smaller than T_K4_ (System A1) one observes an “unmodified” state for transcription levels between T_K27_ and T_K4_. In this state the nucleosomes associated with the gene promoter are neither H3K4me3 nor H3K27me3 modified. Increasing the transcription in this case, the promoter modification state will change from “H3K27me3” to “unmodified” to “H3K4me3”. In the opposite case, where T_K27_ is larger than T_K4_ (System A2), one observes a “bivalent” state for transcriptional levels between T_K4_ and T_K27_. In this state, the nucleosomes associated with the gene promoter are H3K4me3 and H3K27me3 modified.

Which of these cases is actually realized for a particular gene depends, among others, on the promoter accessibility for HMTs. Thus, system A1 (Figure 2A, thin lines) can be changed to system A2 (Figure 2A, thick lines) just by assuming a more open chromatin state (see: Appendix A). Accordingly, our model predicts that cells with an open chromatin state, such as embryonic stem cells (ESC), will have more bivalent genes than cells with more condensed chromatin, in agreement with experimental observations [24]. Another way to induce bivalent states is increasing the cooperativity of HTM binding and thereby inducing bi-stable states of the both histone modifications (Figure 2B, regions I and III, [3]). In the System A1 (Figure 2A), such an increase of the cooperativity switches the unmodified state, seen for transcriptional levels between T_K27_ and T_K4_ into a bivalent state (Figure 2B, region II). Our further studies are focused on systems with high cooperativity of HTM binding.

### 3.2. Balance between Histone Modification States (the Histone Modification Machinery)

In our model each value of the H3K4me3 modification level (m_4_) and the H3K27me3 modification level (m_27_) is associated with a well-defined transcription level *T*_1_(m_4_) and *T*_2_(m_27_), respectively, via a self-consistent equation. In contrast, due to potential bi-stability of the modifications, a specific transcription level can be associated with either one or three different modification levels (compare: Figure 2). The explicit functions *T*_1_(m_4_) and *T*_2_(m_27_) used in our model are given in the Appendix A. All modification states, i.e., all pairs {m_4_*, m_27_*}, which can be realized given a particular organization of the histone modification machinery, need to solve the equation:

(1) $$T_{1}\left(m_{4}*;\{X\}\right)=T_{2}\left(m_{27}*;\{X\}\right)$$

**Figure 3:**
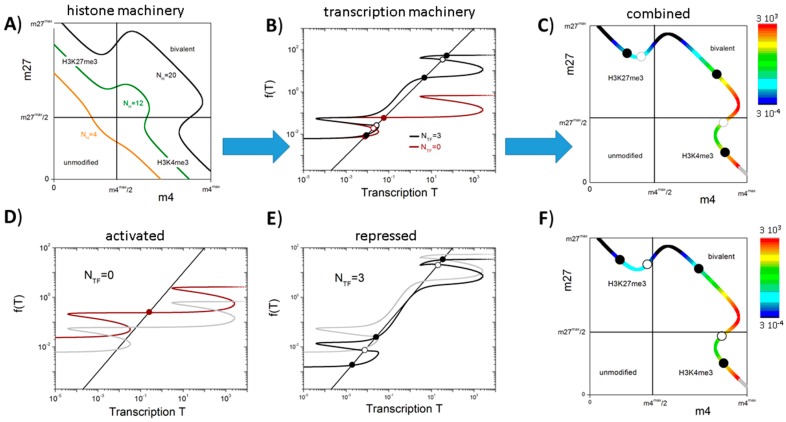
The bivalent state in MV1: (**A**) Shown are the possible pairs {m_4_*, m_27_*} applying parameter set {X} for different numbers (N_H_ = 4 (orange), 12 (green), and 20 (black)) of the cooperative nucleosomes associated with the promoter. The range of m_4_ and m_27_ is exponentially spread approaching its maximum and minimum values (see: Appendix A). The boxes indicate the specific modification states; (**B**) Transcription states derived applying parameter set {Y} for N_H_ = 20 without (brown: N_TF_ = 0) and with (black: N_TF_ = 3) transcriptional auto-feedback. The solutions are given as the intersections of the functions g with the function *y* = *x* (dots); (**C**) Solutions of Equations (1)–(3) in the {m4, m27} space. The solid (open) dots on the curve indicate the stable (unstable) fix points for N_TF_ = 3. The transcriptional level of all other pairs is color coded; (**D**) Transcriptional activation (brown line, F_TF_ = 4); and (**E**) repression (black line, F_TF_ = 1/4) changes the solutions quantitatively as well as qualitatively. (**D**,**E**) References (F_TF_ = 1) are shown as grey lines; (**F**) Solutions of Equations (1)–(3) and fix points for the system shown in (**E**).

Thereby, the solutions depend on the parameter set {X}. Among these parameters are the binding energies of the HMTs, the histone (de-)modification rates and the number N_H_ of cooperative nucleosomes associated with the gene promoter. Examples of the set of possible modification states {m_4_*, m_27_*} are shown in Figure 3A for genes with different numbers of cooperative nucleosomes N_H_. For N_H_ > 5, bi-stable regions for m_4_ and m_27_ are induced (compare: Figure 2B). Thus, for a limited range of m_4_ (m_27_) three possible pairs {m_4_*, m_27_*} exist for each value of m_4_ (m_27_). Under the same conditions, a range of bivalent states becomes manifest that extents with increasing N_H._ The full parameter sets {X} applied in these examples are given in Table A1.

### 3.3. Transcription Controls Epigenetics (the Transcriptional Machinery)

According to the assumptions described above, the histone modification machinery defines all possible modification states that potentially can be realized. Which of them actually become realized is defined by the transcriptional machinery. The fix points of the modeled regulatory circuit {m_4_^#^, m_27_^#^, *T*^#^} are the solutions of the equation:

(2) $$\mathrm{dT}/\mathrm{dt}=f\left(m_{4}*,m_{27}*,T;\{Y\}\right)=0$$

Here, dT/dt is the time derivative of *T* and {Y} refers to the parameter set describing the transcriptional machinery. Among the parameters of {Y} are the maximum promoter activity of a gene and the effective transcript degradation rate. In case a gene encodes a TF that auto-activates itself—which is observed for several key developmental or differentiation regulators, such as the TFs PU.1 or ASCL2 [25,26]—the parameter set {Y} contains also the parameters describing this kind of feedback; as e.g., the number N_TF_ of TF binding sites contained in the gene promoter that are bound by the TF encoded by the gene itself.

As m_4_ and m_27_ depend on *T*, solving Equation (2) in general requires solving a self-consistent equation:

(3) $$T^{\#}=g\left({m_{4}}^{\#},{m_{27}}^{\#},T^{\#};\{Y\}\right)$$

Details about the functions g applied in our study are given in the Appendix B. Figure 3B shows typical solutions of Equations (1)–(3) for different degrees of auto-activation (N_TF_ = 0 and 3). The gene without auto-activation (N_TF_ = 0) possesses three transcriptional states; two of them being stable and one being unstable. The stable, low expression state is associated with an H3K27me3 state and the intermediate expression state with a bivalent one. Introducing auto-activation (N_TF_ = 3) enables a third stable state—a high expressing H3K4me3 state—and increases the expression level of the bivalent state. Figure 3C shows the localization of the obtained solutions and their transcriptional level in the {m_4_, m_27_} space. It can be seen that: (i) H3K27me3 states are always related to low gene expression; (ii) the H3K4me3 states are associated with high gene expression; and (iii) the bivalent states show a broad range of intermediate expression values. For the chosen parameter set {X}, there exists no stable unmodified state independent of the chosen parameter set {Y}. The set {Y} applied in these examples is given in Table A2.

The described circuit can control transitions between regulatory states characterized by different histone modification levels and, thus, transcriptional activities. Such transitions can be induced, e.g., by a TF network that is linked to the gene of interest [10]. The modification states and the transcriptional activity of such a “network-driven” genes are shown in Figure 3D–F. The gene that is solely activated by the TF network (N_TF_ = 0, F_TF_ = 4, Figure 3D) has only a single stable bivalent state. However, small changes of its transcriptional activity can induce state transitions. In particular, an increase (decrease) of less than 35% of the transcriptional activity through the background TF network enables the gene to switch into a monovalent H3K4me3 (H3K27me3) state. This option does not exist for the reference gene (F_TF_ = 1), which can only switch between a bivalent and a monovalent H3K27me3 state. The gene with auto-feedback that is repressed by the background TF network (N_TF_ = 3, F_TF_ = 1/4, Figure 3E) possesses a bivalent state with low transcriptional activity, while the reference system (F_TF_ = 1) shows a bivalent state with high activity. Thus, switches into H3K4me3 and H3K27me3 states will occur with different frequencies. Figure 3F shows the localization of the fix points following gene repression and their transcriptional level in the {m_4_, m_27_} space. Remarkably, for genes that are slightly less repressed, two stable bivalent states can be observed, i.e., multiple bivalent states can exist for a single gene that is embedded in a larger regulatory network structure.

Changes between the stable states require fluctuations of the histone modification level. Details on the stability of the fix points of the system under permanent histone (de-)modification are given in Appendix C. These results demonstrate that, for the applied parameter sets, the bivalent states are more or less long term stable. Hence, cell division-related fluctuation, i.e., cell division-related dilution of modified nucleosomes, are required to resolve the bivalent states. This effect was considered in the following stochastic simulations.

### 3.4. Histone Modification Can Instruct Gene Expression

Epigenetic lineage priming and specification have been described for different stem cell systems including the hematopoietic system [27]. Thereby, resolution of bivalent chromatin states of TFs has particularly been associated with T-cell specification. The maybe best analyzed example is *GATA3* [7,27,28]. The promoter of this gene is bivalent modified in hematopoietic stem cells (HSCs), multipotent (MPPs) and common lymphoid progenitors (CLPs) as well as in B-cells and becomes activated, i.e., it loses H3K27me3, in T-cells. In contrast, *GATA3* expression becomes repressed, i.e., its promoter loses H3K4me3, in common myeloid (CMPs), granulocyte-macrophage (GMPs), and megakaryocyte-erythroid Progenitors (MEPs). Moreover, *GATA3* is auto-activated [29] and the promoter DNA of *GATA3* remains unmethylated in all analyzed cell types [28]. Thus, resolving the bivalent mark at nucleosomes associated with the *GATA3* promoter during proliferation can serve as an example of an instructive role of histone modifications regarding gene expression. If the promoter loses the H3K27me3 marks, *GATA3* expression becomes up-regulated. Up-regulation of *GATA3* increases the potential to specify into T-cell lineages [30]. If the promoter loses the H3K4me3 marks, *GATA3* expression becomes down-regulated and the cell starts to specify more likely into the myeloid lineage [31].

**Figure 4:**
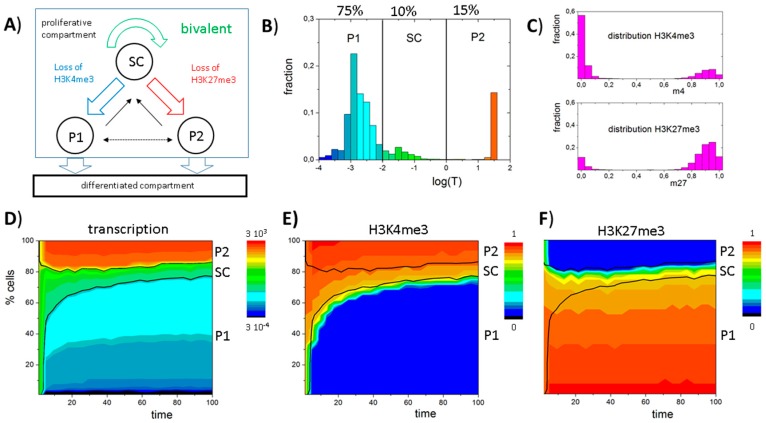
An instructive role of histone modification for *GATA3* expression heterogeneity: (**A**) Sketch of the model of GATA3 heterogeneity. All cells proliferate. Expansion of the compartment is balanced by loss of pro-myeloid progenitors (P1) or pro-lymphoid progenitors (P2) cells that change into a differentiated compartment; (**B**–**F**) Simulation results for a system with stable self-renewal of the bivalent GATA3 state (SC); (**B**) Simulated distribution of *GATA3* expression in the proliferative compartment at *t* = 100 (see: **D**–**F**). All three states, SC, P1 and P2, are observed; (**C**) Bimodal distributions of H3K4me3 and H3K27me3 states are observed within the population; (**D**,**E**) Time dependent composition of the population as seen for: transcription (**D**); H3K4me3 (**E**); and H3K27me3 (**F**) modification (color coded). Shown are cell numbers averaged over 50 simulations with 100 cells each. Black lines separate the cell types based on transcription levels: P1 < 0.01 ≤ SC ≤ 1 < P2.

In order to study the establishment and long-term maintenance of such a heterogeneous cell population, we translated the experimental findings on a GATA3-dependent lineage specification into a general model. As illustrated in Figure 4A, the model accounts for three different cell types that are defined by their histone modification state: bivalent stem cells (SC), H3K27me3-monovalent, pro-myeloid progenitors (P1), and H3K4me3-monovalent, pro-lymphoid progenitors (P2). Furthermore, we assumed that all cell types proliferate with an average cell cycle time τ_0_ of 2.5 time steps (250 simulation steps). Subsequent to each cell division P1 or P2 cells leave the system providing space for the daughters.

Starting from a homogeneous cell population of bivalent SCs, over time a heterogeneous, albeit dynamically stabilized cell population is established. This heterogeneity inherently emerges due to a random loss of H3K4me3 or H3K27me3 modifications in individual cells after cell division, which leads to bimodal H3K4me3 and H3K27me3 distributions as shown in Figure 4C. As transitions from the bivalent to a monovalent histone modification state are directly linked to changes in gene expression, the different cell types can also be distinguished based on their transcriptional activity (Figure 4B). In the model, bivalent SCs are characterized by an intermediate transcription (0.01 < *T* < 1), while P1 cells express low levels (*T* < 0.01) and P2 cells high levels (*T* > 1) of GATA3 (Figure 4B) closely mimicking experimental results. In contrast to typical flow cytometry measurements, which can only provide a snapshot of the population heterogeneity, computer simulations allow monitoring the dynamic changes of transcriptional (Figure 4D) and epigenetic states (Figure 4E,F) over time. It can be seen that the heterogeneity of the cell population remains long-term stable.

Systematic imbalance of the expression states of *GATA3* can lead to disease. For example, loss of *GATA3* induces B cell lymphoma [32]. This can be due to changes in the histone modification machinery [33]. Examples of related simulations are given in the Appendix D (Figure A4).

### 3.5. DNA Methylation Destabilizes Bivalent States

Thus far, we have neglected DNA methylation. However, transcription of many genes has been demonstrated to be affected by DNA methylation. In particular, methylation of CpG-rich promoters is frequently accompanied by repression of the associated gene [16]. Moreover, age-related changes in DNA methylation can be predicted by histone modification states in young individuals [34]. Thus, we considered effects of promoter DNA methylation on bivalent histone modification in a second model version (MV2).

In this setting, we describe changes of the fraction m_DNA_ of methylated CpGs at the gene promoter, neglecting effects of stochastic methylation of individual CpGs [35]. As consequence of the interactions between histone modifications and DNA methylation (Figure 1B), all possible modification states of the gene, i.e., all triples {m_4_*, m_27_*, m_DNA_*}, need to solve, beside Equations (1)–(3), the equation:

(4) $${\tau \mathrm{dm}}_{\mathrm{DNA}}/\mathrm{dt}=H\left(m_{4}*,m_{27}*,m_{\mathrm{DNA}}*;\{Z\}\right)=0$$

where dm_DNA_/dt is the time derivative of m_DNA_ and {Z} refers to the parameter set describing the DNA methylation machinery. This set includes the probability of de novo DNA methylation D_novo_ and the probability of maintaining DNA methylation D_main_. The parameter set {Z} is given in Table A3. A general difference compared to the system without DNA methylation is that the two histone states are now linked not only via transcription but also via DNA methylation. Details can be found in Appendix E.

**Figure 5:**
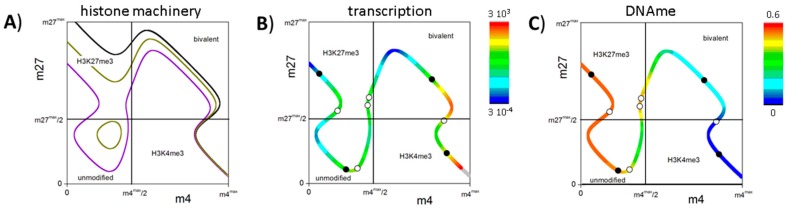
The bivalent state in MV2: (**A**) Shown are all possible modification states applying the parameter sets {X} and {Z}. Increasing de novo DNA methylation probabilities D_novo_ (black: 0, dark yellow: 0.1, violet: 0.3), unmodified states become possible and bivalent states move to lower modification levels; (**B**) Shown is the transcription associated with the modification states; and (**C**) the DNA methylation level of the CpGs of the associated promoter for D_novo_ = 0.3. Values are color coded. The solid (open) dots on the curve indicate the stable (unstable) fix points of the system observed for parameter set {Y}.

Figure 5A shows all possible pairs {m4*, m27*} for such a regulatory system, using the same parameter set as in the unmethylated system (Figure 3A–C) for a fixed number of cooperatively acting nucleosomes (N_H_ = 20). It can be seen that with increasing de novo DNA methylation activity unmodified states become possible, where the nucleosomes carry neither H3K4me3 nor H3K27me3. For the system with a de novo DNA methylation probability of 0.3, four stable states are observed (Figure 5B,C); among them, two intermediate expressing states that are distinguished by their DNA methylation level. The bivalent state shows low, while the unmodified state shows high methylation.

**Figure A2:**
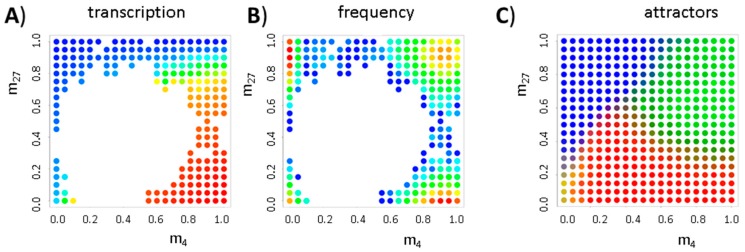
Effects of stochastic histone (de-)modification II. Shown are results for the simulated distribution of histone modification states for a system with transcriptional auto-activation (N_TF_ = 3). In contrast to Figure A1D–F, DNA methylation changes are considered (D_novo_ = 0.3). The color codes (blue: low, red: high) for transcription of the gene (**A**); the frequency of the states (**B**); and the basins of attraction (**C**) are as follows: blue, H3K27me3; green, bivalent; red, H3K4me3; and yellow, unmodified. Introducing DNA methylation, the basin of attraction of the attractor of the bivalent state shrinks (compare Figure A1F).

**Figure A4:**
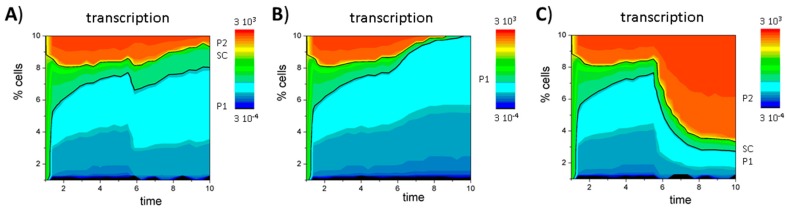
Epigenetic de-regulation of the GATA3 circuit. Shown are simulation results demonstrating the dependence of the systems composition on the absolute values of the (de-)modification rates k_mod_ and k_de_. At time *t* = 5, these rates are decreased: (**A**) for both modifications; (**B**) for H3K4me3 only; and (**C**) for H3K27me3 only, by a factor of 10, while their ratio C^K^ remains conserved. All other parameters and color code are set as in Figure 4.

As discussed above, state fluctuations are required to switch between the stable modification states. In simulations, we observed that DNA methylation reduces the stability of the bivalent state against fluctuations. The basin of attraction of the bivalent state shrinks and the state becomes less frequent populated (see: Appendix C, Figure A2). Thus, in the model, DNA methylation has similar effects on bivalent states as chromatin compaction (compare: Figure 2A and Appendix A) but can occur independently. In cells these events are often linked [24].

### 3.6. A Model of Blast Formation during AML

Besides GATA3, PU.1 is a second gene encoding a major TF responsible for specification into either the myeloid or the lymphoid lineage. In HSCs and MPPs the *PU.1* promoter is associated with H3K4me3 only and accordingly the gene is expressed [27]. In myeloid progenitors *PU.1* expression becomes further up-regulated, while in T-cell progenitors the gene becomes down-regulated [36]. In T-cells, the promoter loses H3K4me3 [27] and becomes methylated [37]. *PU.1* can become auto-activated because it encodes a TF that binds back to its promoter [25]. This binding was suggested to result in recruitment of TET2-proteins and subsequently in active demethylation of the promoter in all cells where *PU.1* is activated [38]. Strikingly, in myeloma cells, *PU.1* becomes down-regulated and methylation of the *PU.1* promoter is found similar to T-cells [39]. Based on these observations, we suggest a mechanistic model of *TET2* mutation associated AML.

**Figure 6:**
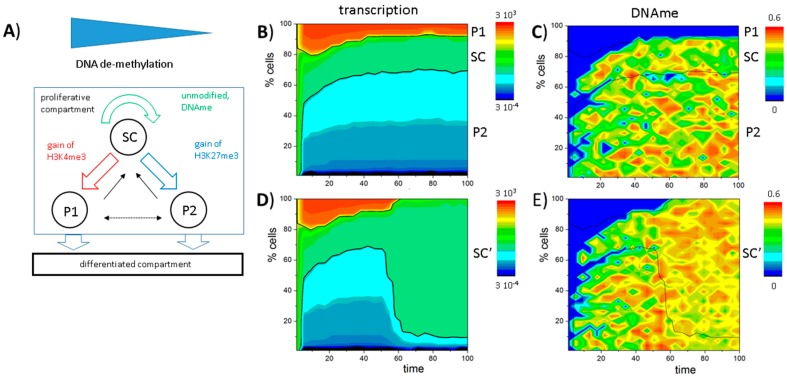
Model of blast formation: (**A**) Sketch of the assumed model of PU.1 regulation. Note that DNA demethylation is expression dependent. As in the GATA3 model, only P1 and P2 cells exit the proliferative compartment; (**B**,**C**) *PU.1* regulation in undisturbed cells. Stable high expression (P1) is enabled by permanent active DNA demethylation. In P2 cells *PU.1* is repressed by H3K27me3 and DNA methylation; (**D**,**E**) *PU.1* regulation in blast cells. Loss of active DNA demethylation (at *t* = 50) compromises *PU.1* activation and leads to expansion of SC-like blasts SC’; (**B**–**E**) Shown are cell numbers averaged over 50 simulations with 100 cells each. Colors encode expression (**B**,**D**) or DNA methylation (**C**,**E**). Black lines separate the cell types based on transcription levels: P1 < 0.01 ≤ SC ≤ 1 < P2.

Figure 6A shows a sketch of a model of undisturbed PU.1 regulation (SC: unmodified, multi-potent progenitors: 0.01 < *T* < 1, P1: H3K4me3-monovalent, pro-myeloid progenitors: *T* < 0.01, P2: H3K27me3-monovalent, pro-lymphoid progenitors: *T* > 1), which considers both DNA methylation and demethylation. Thereby, DNA demethylation occurs permanently and its rate is assumed to be proportional to the expression of the *PU.1* gene as suggested by the experimental finding mentioned above (see also: Appendix E). As in the GATA3 model, we assume that all cell types proliferate and that subsequent to each cell division (if available) only P1 or P2 cells are transferred into a differentiated compartment, providing space for the daughters.

**Figure A5:**
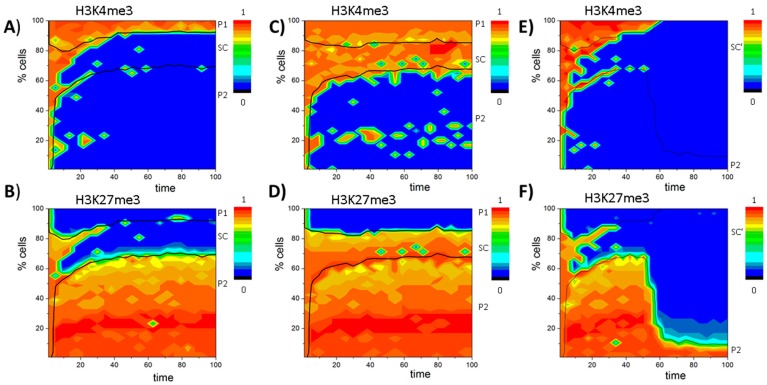
Histone states of the PU.1 circuit: (**A**,**B**) simulation results on the histone states of the system shown in Figure 6 ((**A**) H3K4me3; and (**B**) H3K27me3); and (**C**,**D**) results for the same system assuming an increase of the cell cycle time τ = 10τ_0_ ((**C**) H3K4me3l and (**D**) H3K27me3) enabling re-establishment of the bivalent state. The time is scaled to show the same number of cell cycles, as in (**A**–**F**). Loss of function of the DNA demethylation in the blast system (at *t* = 50) leads to expansion of the unmodified state ((**E**) H3K4me3; and (**F**) H3K27me3). Black lines separate cell types. All values are color coded.

**Figure A6:**
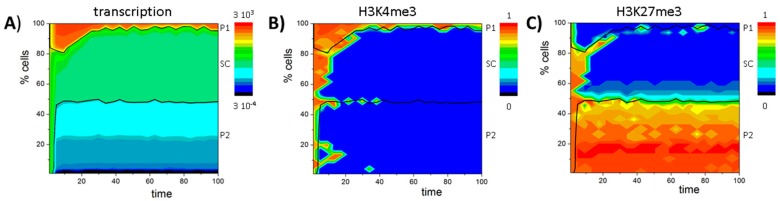
Expansion of the SC population following accelerated proliferation. Shown are simulation results for the PU.1 circuit assuming an acceleration of the proliferation τ = 0.6τ_0_: (**A**) PU.1 expression; (**B**) H3K4me3; and (**C**) H3K27me3. The number of unmodified cells increases similar to the blast system. The time is scaled to show the same number of cell cycles as in Figure 6 and Figure A5A,B. All values are color coded.

Simulation results of that model are shown in Figure 6B,C. In contrast to the GATA3 model, SCs are characterized by an unmodified and partially DNA methylated state. A bistable state is not observed because both H3K4me3 and H3K27me3 have been destabilized by DNA methylation. In committed cells, *PU.1* becomes either up-regulated (P1) following gain of H3K4me3 or down-regulated (P2) following gain of H3K27me3 (Appendix F, Figure A5A,B). Thereby, gain of modification is enabled as a consequence of the ongoing active DNA demethylation between two cell division events. Thus, it depends on the cell cycle time τ. Increasing τ, such that DNA demethylation is effective even for slowly demethylating promoters of repressed genes (compare: Appendix E, Equation (A9)), the bivalent state becomes stabilized (Appendix F, Figure A5C,D). In contrast to the GATA3 system, an expansion of the SC state is observed, accelerating and not decelerating proliferation (Appendix F, Figure A6).

To model blast formation, we assume that active DNA demethylation is no longer functional, e.g., according to mutations in the gene encoding *TET2* [40]. Consequently, the promoter of the *PU.1* gene, as other promoters being under the control of active DNA demethylation by TET2-proteins, becomes methylated independent of the expression of *PU.1*. This results in complete de-modification of the *PU.1* promoter also in cells where *PU.1* is expressed under normal conditions. The affected cells express *PU.1* at an intermediate level and thus are assumed to remain in the expansive niche (see cell type definition). This copes the phenotype known from so-called “blast cells” (SC′), which show *PU.1* hypermethylation and are proliferative active [41]. Simulations of the system (Figure 6D,E and Appendix F, Figure A5E,F) actually show that nearly all cells become fixed in the unmodified and DNA methylated state immediately after the *TET2* mutation is induced (*t* = 50).

The system can be re-transformed by external triggered DNA demethylation but will fall back into the blast system in case this trigger is taken away. In contrast to recruited DNA demethylation, external triggered DNA demethylation (e.g., applying DNMT1 inhibitors) will affect all expression states in the same way and thus induces the bivalent and not the unmodified state. Under such forced DNA demethylation the *PU.1* expression might be similar to the un-mutated system, but the specification of P1 and P2 is enforced by higher and not by lower cell division frequency (see discussion).

## 4. Discussion

Transcriptional feedback loops that enable switches between gene expression states have been identified as basic motifs of gene regulatory networks [42]. Combination of feedback mechanism allows triggering decision processes between alternative gene expression programs. Histone modifications have been originally thought to stabilize the so achieved states [43]. Nevertheless, bivalent modifications have early been implicated also in decision making regarding developmental and stem cell differentiation processes [6]. Here, we provided a mechanistic model on how they might confer their regulatory capacity. In our model, both the activity of TFs as well as cell division events can change the state of bivalent modified promoters. Accordingly, circuits of bivalent genes can be considered as responsive or instructive, respectively. The latter because cell division can not only induce switches between histone modification states [3], but can also robustly trigger cell intrinsic decision processes. In contrast to extrinsically triggered decisions, these decisions do not require a heterogeneous or fluctuating environment. They only require random distribution of histones from the mother onto the daughter cells. Thereby, cell cycle time affects the proportions of decision making. Consequently, the model predicts proliferation activities to be essential for gene expression heterogeneity. Interestingly, a recent report on bivalent genes suggests that in fast proliferating cells, as ESCs, histone modification is under cell cycle control [44].

We focused on the case where the nucleosomes associated with the gene promoter carry both H3K4me3 and H3K27me3. ChIP-seq measurements alone do not provide this information and cannot distinguish this case from a case where a fraction of cells carries one and the complementary fraction the other modification. However, such kind of heterogeneity might be equally important for lineage specification and developmental processes as shown for H3K27me3 heterogeneity [45].

Our theoretical studies build on previous results of our group on the dynamics of histone modifications that are set by HMTs, being part of protein complexes that can read the modification. The gene promoters bound by these HMTs can assume bi-stable modification states capable of encoding a memory. Similar approaches have been published for the first time by Dodd et al. [46,47]. More complex models of cooperative pattern formation of histone modifications have been suggested by Anink-Groenen et al. [48]. Here, we studied a model combining histone modifications that can activate and repress gene transcription. Such a circuit has also been approached mathematically by Ku et al. but without a direct link between histone modification and transcription [49]. The regulation proposed by our model defines a set of histone modification states that can be realized in general, while the specific coupling of the gene’s transcription to the modifications specifies the states that are actually realized under given conditions. Thus, our approach allows separating the properties of the regulatory circuit that rely on the histone modification machinery from those relying on the TF-network. Thereby, existence of bivalency depends, among others, on the accessibility of the promoter.

In our model, DNA methylation de-stabilizes bivalent states reducing the effective binding energy of the HMTs to the promoter, i.e., reducing its accessibility. Such de-stabilization can induce an unmodified histone state. We have shown that introducing active DNA demethylation, gene expression heterogeneity can also result from this unmodified state as a consequence of successive DNA demethylation during the cell cycle; increasing the accessibility again. Assuming that the effectiveness of this process depends on the expression of the gene, first activated and at later times also repressed genes become DNA demethylated. Thus, also in this case, cell cycle time affects gene expression heterogeneity. Consequently, the response of gene expression heterogeneity on changing proliferation activities might dependent of the promoter methylation dynamics of the gene under consideration. Recent experimental findings suggest that they show large clonal differences [35].

We applied our circuits to explain gene expression heterogeneity in hematopoietic cells. The examples provided describe qualitative features of expression heterogeneity of two genes encoding basic TFs of hematopoietic lineage specification, *GATA3* and *PU.1*. Activation of these genes is known to be associated with an increased potential to specify into lymphoid and myeloid lineages, respectively. These models may not qualify to explain the entire specification process into these two lineages, as e.g., [36], but they provide, for the first time, clear hypotheses on how histone modification might be involved. Thereby, the models are consistent with different experimental findings on histone modification profiles of HSCs and their progeny. Formally, they could be used to fit fluorescence-activated cell sorting (FACS) data on the decision process, but this would overstress their potential at the current state of the art.

The two examples (*GATA3*, *PU.1*) were chosen as closely related as possible, sharing all parameters of the histone and transcriptional machinery. This allows us to demonstrate that the introduction of DNA methylation can impair one regulatory principal while potentially introducing a “complementary” one. Thus, tight regulation of DNA methylation is an essential mechanism not only controlling bivalency but also a regulatory mechanism during lineage specification in general.

Our AML model bases on break down of an active lineage specification mechanism. Under normal conditions, self-renewal of the stem cells (SC) requires DNA methylation, in agreement with experimental findings on HSC self-renewal [50], and specification of P1 requires active DNA demethylation. Loss of demethylation, i.e., of the active lineage specification mechanism, induces hypermethylation of the *PU.1* promoter and a differentiation block in agreement with experimental findings in AML [39] as well. Such loss of demethylation can be expected in about 28% of AML cases, where either the *TET2* gene or the isocitrate dehydrogenase 1/2 (*IDH1/2*) genes are mutated [51]. Beside *PU.1* many other demethylation targets are affected in these cases. However, a similar affect can be expected by down-regulation of *PU.1* alone, as TET2 is recruited to *PU.1* binding sites. Consistently, a moderate down-regulation of *PU.1* indeed is sufficient to induce AML in mice [52]. Moreover, in about 26% of AML cases mutations of DNMT3a have been found [51]. Changes in DNMT3a function will affect the DNA methylation efficiency and thus also affect the outcome of our model. However, we have shown that lineage specification in the PU.1 system can be repressed simply by accelerating proliferation. This agrees with experimental findings that DNMT3a-mediated promoter hypermethylation is rather a consequence of AML progression, in particular of enforced proliferation, than the origin of AML [53].

We already mentioned that, analog to *PU.1*, also systematic imbalance of the expression states of *GATA3* can lead to tissue transformation and that loss of *GATA3* has been found to induce B cell lymphoma [32]. In the last years, it has been shown that *PU.1* and *GATA3* interact to control the myeloid-lymphoid switch [31]. Thus, the models introduced here can be considered as basic modules of a more complex model of this essential switch in hematopoietic lineage specification.

## 5. Conclusions

In agreement with experimental observations, our model suggests that bivalent states are fostered by open chromatin and a high degree of cooperative binding of H3K4me3 and H3K27me3 HMTs. Loss of bivalency due to promoter de-modification subsequent to cell division can instruct gene expression. Accordingly, major effectors of gene expression heterogeneity are the kinetics of histone modification reactions and the cell cycle time. Decreasing the cell cycle time de-stabilizes the bivalent state. This could be experimentally tested, e.g., for the *GATA3* promoter.

Such destabilization can also be achieved by DNA methylation, which potentially replaces bivalent by unmodified states. In such a case, active DNA demethylation during the cell cycle can instruct gene expression similar to loss of bivalency after cell division. In addition, here, emerging gene expression heterogeneity depends on the time scale of histone modification reactions and on the cell cycle time. We suggest that this kind of regulation is impaired during AML blast formation following loss of function mutation of the TET pathway.

Computational models of epigenetic regulation of transcription enable testing hypotheses on the interactions between different (activating and repressive) epigenetic marks. Thus, they can support a mechanistic understanding of chromatin dynamics and its impact on decision processes during development and lineage specification.

## Figures and Tables

**Figure A1:**
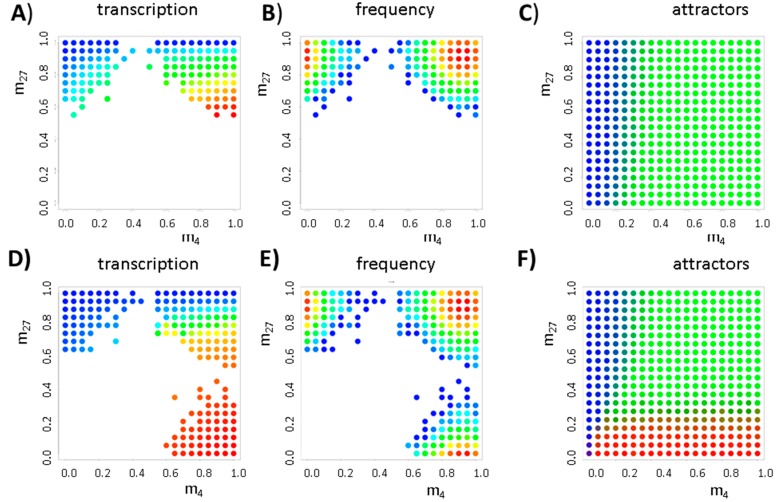
Effects of stochastic histone (de-)modification I. Shown are results for the simulated distribution of histone modification states for a system: without (**A**–**C**) (N_TF_ = 0); and with (**D**–**F**) (N_TF_ = 3) transcriptional auto-activation. The color codes for transcription of the gene (**A**,**D**); and the frequency of the states (**B**,**E**) are as follows: blue, low; and red, high. All N_H_^2^ (400) possible initial states {m_4_, m_27_} have been considered in 100 realizations; (**C**,**F**) The basins of attraction separate the fix points (blue: H3K27me3, green: bivalent, red: H3K4me3).

**Figure A3:**
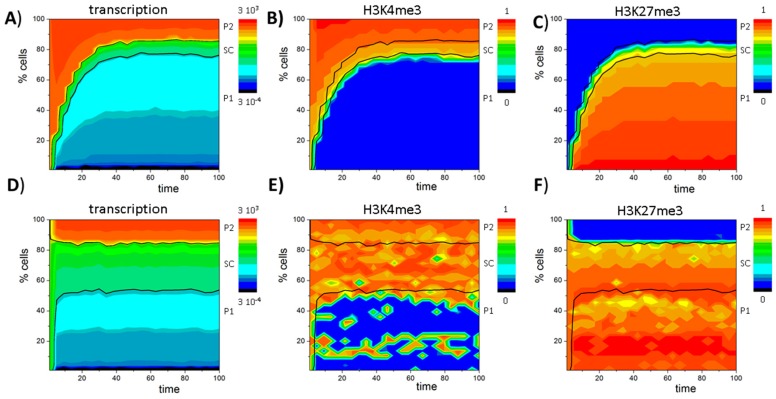
Regulatory states of the GATA3 circuit. Time dependent composition of the system, as seen for: transcription (**A**,**D**); H3K4me3 (**B**,**E**); and H3K27me3 (**C**,**F**) obtained in simulations with different initial conditions ((**A**–**C**) P2 cells only, τ = τ_0_) and increased cell cycle time ((**D**–**F**) τ = 10 τ_0_). The values are color coded. Black lines separate SC from P1 and P2 cells. In (**D**–**F**), the regulatory states have been recorded every 1000th simulation steps to show the same number of cell cycles as in Figure 4D–F.

## Abbreviations

AML	acute myeloid leukemia
HMT	histone methyltransferase
DNMT	DNA methyltransferase
TF	transcription factor
MV1	model version 1
MV2	model version 2

## Appendix A. Model of Histone Modification

### Regulation of Histone Modification

The binding probabilities of the HMTs, θ^K^ (K = 4, 27), are calculated assuming a positive feedback between the presence of the histone mark K and the recruitment of its HMTs. Such a feedback has been demonstrated for both H3K4me3 and H3K27me3 [1]. According to this assumption, the binding probability of the HMT of histone mark K depends on the fraction m_K_ (K = 4, 27) of modified nucleosomes of the N_H_ cooperative nucleosomes associated with the promoter and is given by:

(A1) $$\theta^{K}=\frac{1}{1+\exp\left(\varepsilon_{0}^{K}+\left(1-m_{\mathrm{DNA}}\right)\varepsilon_{\mathrm{BS}}^{K}+m_{K}\varepsilon_{\mathrm{HM}}^{K}N_{H}+\lambda_{K}\log\left(T\right)\right)}$$

where ε_0_^K^ is the ground enthalpy per bound HMT complex and ε_BS_^K^ and ε_HM_^K^ are the free energies of HMT binding to DNA and to histone mark K, respectively. They are specific for histone mark K and are scaled by the Boltzmann unit. A decrease (increase) of ε_0_^K^ describes an increase (decrease) of the promoter accessibility. In this way, System A1 was changed to A2 by decreasing ε_0_^K^ by 2.

For MV1 (stable DNA methylation), the fraction of methylated CpGs of the promoter m_DNA_ is a constant. Setting m_DNA_ = 0, we describe a completely unmethylated promoter DNA. In MV2, m_DNA_ can vary (see: Appendix E). The factor λ_k_ is −1 for H3K4me3 and +1 for H3K27me3. Assuming a dynamic equilibrium between modification and de-modification, Θ^K^ has to solve:

(A2) $$\theta^{K}=\frac{C^{K}m_{K}}{1-m_{K}}$$

where C^K^ is the de-modification constant C^K^ = k_de_/k_mod_; i.e., the ratio between the de-modification rate k_de_ and the modification rate k_mod_ for the respective modification. Using Equation (A2), rearrangement of Equation (A1) according to log_10_(T) yields:

(A3) $$\log_{10}\left(T\right)=\frac{\ln\left(\frac{1-m_{K}}{C^{K}m_{K}}-1\right)-\left(\varepsilon_{0}^{K}+(1-m_{\mathrm{DNA}})\varepsilon_{\mathrm{BS}}^{K}+m_{K}\varepsilon_{\mathrm{HM}}^{K}\right)}{\lambda_{K}}$$

where K specifies the modification, i.e., T = T_1_ (K = 4) and T = T_2_ (K = 27) as described in the text.

Changes of the solutions of Equations (1)–(3) according to parameter changes are often hard to follow in a linear plot of m_27_ versus m_4_. We therefore used a transformation of both variables which spreads their values if they approach their minimum and maximum. We used: m_K_* = 4000 − 500 × ln((m_max_ − m_K_)/m_K_), with m_max_ being the maximum modification level of the respective modification, given by: 1/(1 + C^K^) [3].

**Table A1:** Parameter set {X} of the histone modification machinery. * scaled by the Boltzmann unit.

Parameter	Value	Description
ε_0_^4^, ε_0_^27^	9.0, 10.0	ground enthalpy per bound HMT *
ε_BS_^4^, ε_BS_^27^	−5.0, −5.0	free energy of CpG binding *
ε_HM_^4^, ε_HM_^27^	−0.5, −0.5	free energy of histone binding *
C^4^, C^27^	0.1, 0.1	de-modification constant
N_H_	4/12/20	number of cooperative nucleosomes

## Appendix B. Model of Transcriptional Regulation

### Regulation of Gene Expression

In the original model, we introduced a double positive feedback between transcription and the activating histone mark H3K4me3. Here, we extended the model by introducing a double negative feedback between transcription and the repressive histone mark H3K27me3 (see text). We assumed the transcription of the gene to depend on modification levels m_4_ and m_27_ of the gene promoter. Accordingly, the transcription of the individual genes is calculated by solving:

(A4) $$\frac{\mathrm{dT}}{\mathrm{dt}}=\frac{P_{\max\left(\left(m_{0}+m_{4}\right)\left(1-m_{27}\right)\right)}}{1+\frac{P_{\max}-1}{F_{\mathrm{TF}}F_{\mathrm{auto}}}}-\delta T$$

where P_max_ is the maximum promoter activity and δ the transcript degradation rate. F_TF_ and F_auto_ are the regulation factors for the TF-network and the auto-regulation of the gene, respectively. The constant m_0_ << 1 ensures that genes can be transcribed with a small rate also if the promoter is devoid of H3K4me3 (m_4_ = 0). Details about the underlying regulatory principles, which are based on thermodynamics [54]. F_auto_ is given by:

(A5) $$F_{\mathrm{auto}}={\left(\frac{1+\mathrm{Texp}\left(\varepsilon_{A}\right)}{1+T}\right)}^{N_{\mathrm{TF}}}$$

where ε_A_ is the decrease of the free energy of polymerase binding to the promoter, following auto-activation of the gene. It is scaled by the Boltzmann unit. N_TF_ is the number of binding sites at the promoter for the TF encoded by the gene. Assuming a dynamic equilibrium of the transcription T, the function g of Equation (3) is given by:

(A6) $$\left(T\right)=\frac{\frac{P_{\max\left(m_{0}+m_{4}\right)\left(1-m_{27}\right)}}{1+\frac{\left(P_{\max}-1\right)}{F_{\mathrm{TF}}F_{\mathrm{auto}}}}}{\delta }$$

**Table A2:** Parameter set {Y} of the transcription machinery. Rates are given in events per simulation step. * scaled by the Boltzmann unit.

Parameter	Value	Description
P_max_	100	maximum transcription rate
δ	1.5	transcript degradation rate
ε_A_	2	free energy of polymerase binding *
F_TF_	4/1/0.25	regulation factor of the TF-network
N_TF_	0/3	number of binding sites for auto-activation

## Appendix C. Stochastic Simulation

In a first series of computer simulations of the model, we enabled changes of the histone modification level with defined rates, k_de_ and k_mod_ (C^K^ = k_de_/k_mod_, K = 4, 27). In this series, we did not consider: (i) dilution of modifications due to cell division events; and (ii) active DNA demethylation. The so-defined stochastic system shows similar solutions as the deterministic one. This is shown in Figure A1 providing simulation results for systems without (N_TF_ = 0) and with transcriptional feedback (N_TF_ = 3). The borders between the attractors of the fix points are clearly separated from the fix points themselves. Thus, at the given level of fluctuations, transitions between the different states are rare, i.e., the states are stable over several cell cycles. Figure A2 demonstrates that DNA methylation destabilizes the bivalent state. For the simulations shown in Figure A1 and Figure A2, the parameter sets of the *GATA3* and *PU.1* system have been applied, respectively.

## Appendix D. The GATA3 Circuit

Simulations of the GATA3 circuit have been performed using the parameter sets {X} and {Y} with N_H_ = 20. As an exception, ε_HM_^K^ was changed to 0.55 for K = 4 and 27 in order to stabilize the bivalent state. In Figure A3, additional simulation results for system shown in Figure 4 are provided. Figure A3A–C demonstrates that the fix point of P2 is long term stable, as regeneration of the system from P2 cells takes up to 16 cell cycles (40 time steps). Figure A3D–F demonstrates that the population of bivalent cells expands if the cell cycle time τ is increased. Changes of the parameters of the epigenetic machinery lead to changes in the population heterogeneity. Figure A4 shows simulation results for the dependence of the systems composition on the absolute values of the histone modification rates.

## Appendix E. DNA Methylation Model

In order to calculate regulatory states of genes in the presence of DNA methylation, a simple model of DNA methylation was applied. This model considers the processes of maintenance and de novo DNA methylation at CpG rich promoters. Accordingly, the time derivative of the fraction of methylated CpGs within the promoter m_DNA_ is given by:

(A7) $$\tau \frac{{\mathrm{dm}}_{\mathrm{DNA}}}{\mathrm{dt}}=D_{\mathrm{novo}}h\left(m_{4},m_{27}\right)\left(1-m_{\mathrm{DNA}}\right)-\left(1-D_{\mathrm{main}}\right)m_{\mathrm{DNA}}$$

where D_main_ and D_novo_ are the maintenance and de novo DNA methylation probabilities, respectively. The function h depends on the histone modification state of the nucleosomes associated with the promoter. It is given by:

(A8) $$h=\frac{1+\exp\left(-D_{\mathrm{CG}}^{27}m_{27}\right)}{1+\exp\left(D_{\mathrm{CG}}^{4}m_{4}-D_{\mathrm{CG}}^{27}m_{27}\right)}$$

where D_CG_^K^ (K = 4, 27) are constants which describe the impact of histone modification K on binding of the de novo DNMT to the promoter. H3K4me3 methylation suppresses the binding. Thereby, D_CG_^4^ is the free energy change associated with binding at m_4_ = 1 scaled by the Boltzmann unit. Presence of H3K27me3 weakens the effect of H3K4me3, while at m_4_ = 0 it has no effect. Calculating solutions of Equations (1)–(3) in presence of DNA methylation, a stationary state dm_DNA_/dt = 0 is assumed (see Equation (4)). In stochastic simulations of the system, DNA methylation is updated after each cell division only, i.e., the D_main_ and D_novo_ are probabilities per cell division event.

In the model of the PU.1 circuit active DNA demethylation is assumed in addition. According to the experimental finding that binding of the PU.1 protein to the promoter recruits DNA demethylation enzymes (TET-proteins), the rate of (de-)modification is assumed to be *PU.1* expression dependent. Thus, in the simulations, demethylation occurs with a rate:

(A9) $$D_{\mathrm{DE}}=\frac{D_{\mathrm{DE}}^{0}T\delta }{P_{\max}}$$

where D_DE_^0^ is the maximum DNA demethylation rate per simulation step.

**Table A3:** Parameter set {Z} of the DNA methylation machinery. Rates are given in events per simulation step. * scaled by the Boltzmann unit.

Parameter	Value	Description
D_main_	0.8	probability of maintaining DNA methylation
D_novo_	0.0/0.1/0.3	probability of de novo DNA methylation
D_CG_^4^, D_CG_^27^	6, 4	interaction energy between HMTs and DNMTs *
D_DE_^0^	6/2	maximum rate of active DNA demethylation

## Appendix F. The PU.1 Circuit

Simulations of the PU.1 circuit have been performed using the same parameter sets as for the GATA3 circuit. DNA (de-)methylation was enabled applying the parameter set {Z} (see: Table A3).
